# A human case of *Dioctophyma renale* (giant kidney worm) accompanied by renal cancer and a retrospective study of dioctophymiasis

**DOI:** 10.1051/parasite/2019023

**Published:** 2019-04-09

**Authors:** Fengkun Yang, Weizhe Zhang, Baiyan Gong, Lan Yao, Aiqin Liu, Hong Ling

**Affiliations:** Department of Parasitology, Harbin Medical University Harbin Heilongjiang 150081 PR China

**Keywords:** *Dioctophyma renale*, Dioctophymiasis, Humans, Renal cancer

## Abstract

Due to the rarity of human cases and the nonspecific clinical symptoms of dioctophymiasis, *Dioctophyma renale* infection is not well recognized and is easily neglected or misdiagnosed. Recently, we diagnosed a human case of dioctophymiasis accompanied by renal cancer. To enhance the understanding of human dioctophymiasis, this case is presented here, and a retrospective study of this disease was conducted based on relevant papers screened from PubMed and three Chinese databases. In the end, 32 papers describing 37 human cases of dioctophymiasis were assessed. These cases were distributed in ten countries of Asia, Europe, North America and Oceania, with the highest number in China (*n* = 22). The majority of the cases occurred in adults (91.9%, 34/37) and involved the kidneys (83.8%, 31/37). Ectopic parasitism mainly occurred in subcutaneous tissue (83.3%, 5/6). A proportion of 45.9% (17/37) of individuals had a history of eating raw or undercooked fish or frogs. The main clinical manifestations of human dioctophymiasis were loin pain (59.5%) and hematuria (59.5%). All the cases were diagnosed based on the morphological characteristics of eggs or adults in urine or tissue sections. Currently, there is no strictly defined therapeutic approach. This is the first retrospective analysis of human cases of dioctophymiasis. These review data will deepen our understanding of dioctophymiasis and help avoid misdiagnosis in clinical practice.

## Abstract (fr)

En raison de la rareté des cas humains et des symptômes cliniques non spécifiques de la dioctophymiose, l’infection par *Dioctophyma renale* n’est pas bien reconnue et est facilement négligée ou mal diagnostiquée. Récemment, nous avons diagnostiqué un cas humain de dioctophymiose accompagné d’un cancer du rein. Pour améliorer la compréhension de la dioctophymiose humaine, ce cas est présenté ici et une étude rétrospective de cette maladie a été menée à partir de documents pertinents sélectionnés dans PubMed et dans trois bases de données chinoises. Finalement, 32 articles décrivant 37 cas humains de dioctophymiose ont été examinés. Ces cas ont été répartis dans dix pays d’Asie, d’Europe, d’Amérique du Nord et d’Océanie, le nombre le plus élevé étant enregistré en Chine (*n* = 22). La majorité des cas sont survenus chez des adultes (91,9 %, 34/37) et concernaient le rein (83,8 %, 31/37). Le parasitisme ectopique est principalement survenu dans les tissus sous-cutanés (83,3 %, 5/6). Une proportion de 45,9 % (17/37) des personnes avaient déjà mangé du poisson ou des grenouilles crus ou pas assez cuits. Les principales manifestations cliniques de la dioctophymiose chez l’homme étaient les douleurs lombaires (59,5 %) et l’hématurie (59,5 %). Tous les cas ont été diagnostiqués sur la base des caractéristiques morphologiques des œufs ou adultes dans l’urine ou les coupes de tissus. Actuellement, il n’y a pas d’approche thérapeutique strictement définie. Ceci est la première analyse rétrospective de cas de dioctophymiose chez l’homme. Les données de cette revue approfondissent notre compréhension de la dioctophymiose et aideront à éviter les erreurs de diagnostic en pratique clinique.

## Introduction

*Dioctophyma renale* commonly referred to as the “giant kidney worm” is one of the largest parasitic nematodes. Adult worms are found in the kidneys of many flesh-eating mammal species, including humans. Humans acquire infection with *D. renale* mainly by eating raw or undercooked fish or frogs containing infective larvae. Individuals with *D. renale* infection usually have nonspecific clinical symptoms similar to nephritis, mainly including loin pain and hematuria [21]. Most seriously, three fatal cases of dioctophymiasis have been reported in Indonesia, the United States and China [17, 19, 24]. Human cases of ectopic parasitism are reported to occur in subcutaneous nodules and the retroperitoneal cavity [1, 2, 11, 25, 27, 28].

**Figure 1:**
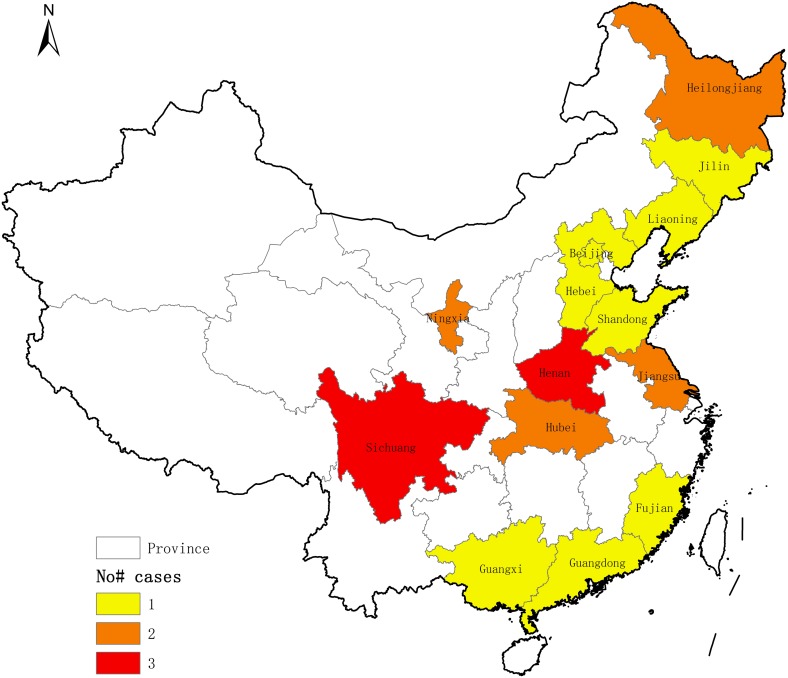
Existence of *Dioctophyma renale* in multiple provinces and municipalities in China. Three, two and one human cases of dioctophymiasis have been reported in provinces filled in red, orange and yellow, respectively.

*Dioctophyma renale* is worldwide in distribution, but rarely causes human infection [4]. To date, human cases have only been reported in 10 countries (Table 1) [1, 2, 4–26, 30, 31, 33–35]. In China, since the first report of human dioctophymiasis in 1981, a total of 21 human cases have been documented, distributing in at least 14 provinces and municipalities [35] (Fig. 1). Recently, a patient suffering from renal cancer expelled at least 15 worms, which were later identified as *D. renale* based on the morphological characteristics of the worms.

**Table 1:** Human cases of *Dioctophyma renale* worldwide published in PubMed and Chinese databases.

Country (case no.)	Age	Gender	No. of worms	Clinical characteristics		Location	Stage used for diagnosis (sample)	Suspected source of infection	References
				Loin pain/Hematuria	Others				
*China*									
Beijing (1)	59	Female	1	+/+		Right kidney	Adult (urine)		Feng [7]
Fujian (1)	30	Female	2	–/+	Urgent urination, abdominal pain, odynuria	Kidney	Adult (urine)		Gong [9]
Guangdong (1)	31	Female	2	+/+		Right kidney	Adult and egg (urine)	Raw fish	Zhang and Zhu [35]
Guangxi (1)	20	Female	1	+/–		Kidney	Adult (urine)	Raw fish and frog	Lei et al. [18]
Heilongjiang (2)	47	Male	5	+/+ Pr	Frequent urination, anemia	Right kidney	Adult (urine)		Liu [20]
	49	Female	15	+/+ Py	Frequent and urgent urination	Right kidney	Adult (urine)	Unboiled water	This study
Hebei (1)	45	Male	1	+/+	Fever	Right kidney	Adult (urine and pelvis)		Gu et al. [10]
Henan (3)	39	Male	7	+/+	Frequent urination, anemia	Right kidney	Adult (urine)	Raw fish, unboiled water	Wang [31]
	32	Female	1	–/–		Kidney	Adult (urine)		Sun et al. [26]
	46	Female	>10	+/+	Abdominal pain	Kidney	Adult (urine)		Peng et al. [22]
Hubei (2)	36	Male	1	+/+		Kidney	Adult (urine)		Zhang and Zhu [35]
	30	Male	1	+/–	Frequent and urgent urination	Right kidney	Adult (urine)	Raw fish	
Jiangsu (2)	31	Female	1	+/–	Fever, frequent and urgent urination, odynuria	Left kidney	Adult (urine)		Zhang and Zhu [35]
	92	Male	Unspecific	+/+		Left kidney	Larva and egg (urine)	Raw fish	Yang et al. [34]
Jilin (1)	55	Male	1	–/– Py	Urgent urination, odynuria	Kidney	Adult (urine)		Jin et al. [15]
Liaoning (1)	67	Female	3	–/+ Pr		Kidney	Adult (urine)	Raw fish	Cui et al. [6]
Ningxia (2)	47	Male	1	–/–		Kidney	Adult (urine)	Raw fish and frog, unboiled water	Qiu et al. [23]
	20	Female	1	+/–	Fever, urgent urination	Right kidney	Adult (urine)	Unboiled water	
Shandong (1)	51	Female	39^c^	+/+ Py		Bilateral kidney	Adult and egg (urine)	Raw fish	Li et al. [19]
Sichuang (3)	9	Male	1	+/+	Anemia	Right kidney	Adult (urine)	Raw fish	Hu [13]
	53	Female	15	–/+ Pr	Abdominal pain	Kidney	Adult (urine)	Raw fish	Yang and Lu [33]
	6	Male	1	+/–	Fever, frequent and urgent urination	Kidney	Adult (urine)		Chen and Liu. [5]
*Other countries*									
Australia (1)	47	Male	–	+/+	Renal colic	Left kidney	Egg (tissue)		Fernando [8]
Greece (1)	39	Male	–	+/–		Right kidney	Adult (tissue)		Katafigiotis et al. [16]
India (2)	35	Male	Unspecific	–/+ Pr	Fever, retention of urine	Bilateral kidney	Adult (urine)	Raw fish	Chauhan et al. [4]
	70	Male	2	–/+	Fever	Right kidney	Adult and egg (urine)	Raw fish	Venkatrajaiah et al. [30]
Indonesia (1)	67	Male	2 + 23^b^	+/+	Abdominal pain, anemia	Left kidney	Adult (urine)	Raw fish, unboiled water	Sardjono et al. [24]
Iran (2)	75	Male		+/+		Right kidney	Adult (pelvis), egg (urine)	Unboiled water	Norouzi et al. [21]
	28	Female	–	–/+	Fever, abdominal pain	Kidney	Egg (urine)		Hanjani et al. [12]
Japan (2)	26	Female^a^	–	–/–	Papule with itching	Thigh	Larva (tissue)		Urano et al. [28]
	44	Male^a^	–	–/–	Papule	Abdominal wall	Larva (tissue)		Tokiwa et al. [27]
Thailand (1)	12	Male	–	–/–	Subcutaneous nodule	Chest wall	Larva (tissue)		Beaver and Khamboonruang [2]
USA (4)	71	Male	>1	–/+	Anemia, weight loss	Left kidney	Adult (urine)	Raw fish	Kuehn et al. [17]
	26	Male	–	–/–	Subcutaneous nodule	Chest wall	Larva (tissue)		Beaver and Theis [1]
	23	Female	–	–/–	Subcutaneous nodule	Abdominal wall	Larva (tissue)	Raw fish	Gutierrez et al. [11]
	50	Male^a^	–	+/–	Fever	Retroperitoneal cavity	Egg and adult (tissue)	Raw fish	Sun et al. [25]
Yugoslavia (1)	44	Male	–	+/+ Py		Bilateral kidney	Adult (tissue), egg (urine)	Raw fish	Ignjatovic et al. [14]

The bars denote negative results; Pr = proteinurina; Py = pyuria.

a Representing patients who became ill in the countries where they lived, including two Chinese patients living in Japan for four and 15 years, respectively and one Chinese patient living in the United States;

b 23, representing 23 fragments or worms expelled in urine;

c 39, representing 39 fragments of worms expelled in urine.

Currently, due to the rarity of human cases of dioctophymiasis, *D. renale* infection is not well recognized and is easily misdiagnosed by clinicians. To enhance the understanding of this parasitic disease and reduce occurrence of misdiagnosis in clinical practice, this case was presented in detail and additionally, a retrospective study of human cases of dioctophymiasis was performed, especially including some case reports published in Chinese which may not be well known.

## Case report

**Figure 2:**
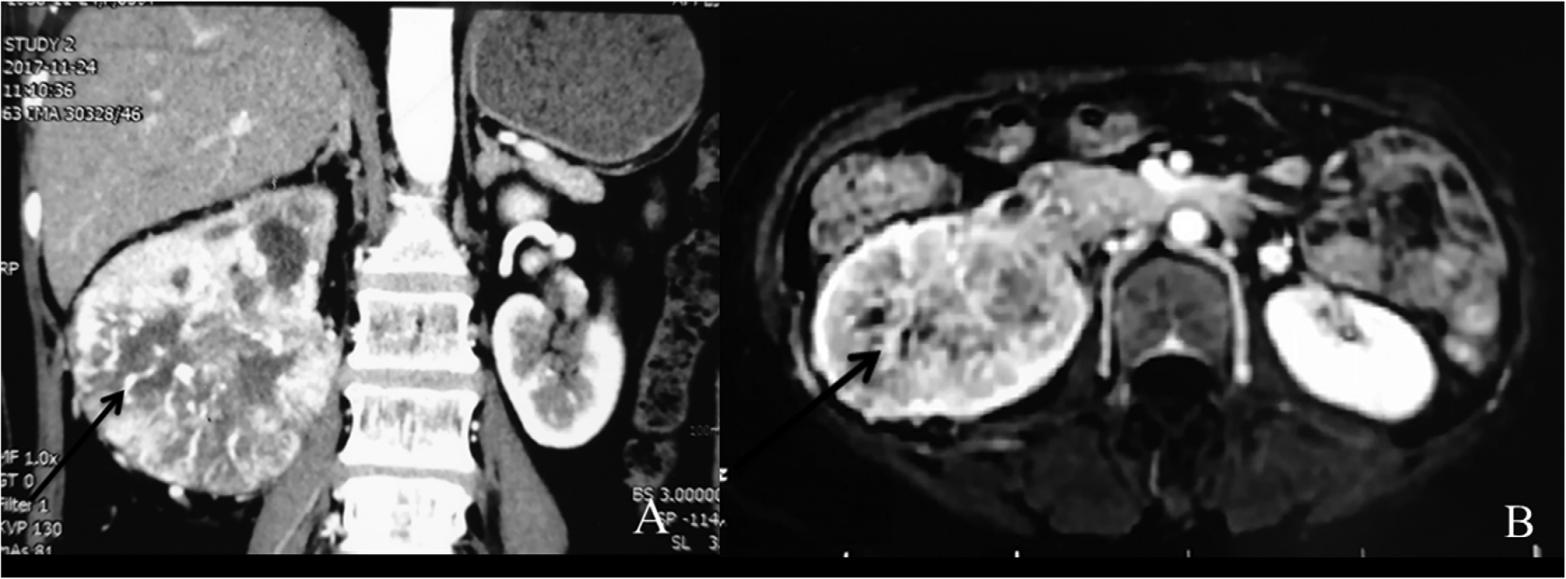
MRI findings and an expelled worm. Axial and coronal MRI revealed that the right kidney was enlarged and severely damaged **(2A and 2B)**.

**Figure 3:**
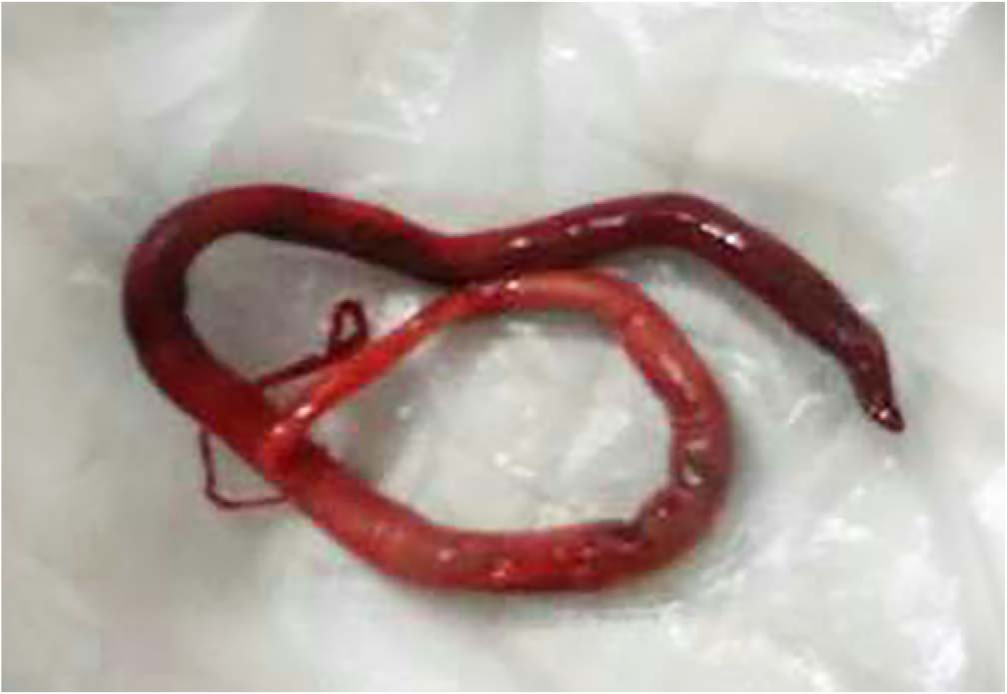
An adult *Dioctophyma renale* worm found in the patient’s urine.

On May 10, 2017, a 49-year-old Chinese woman was admitted to the Third Affiliated Hospital of Harbin Medical University due to gross hematuria for four days, with a presentation of intermittent right loin pain for two years. Besides hematuria, pyuria was found in initial urinalysis. Physical examination showed notable percussion pain over the right costovertebral angle. Magnetic Resonance Imaging (MRI) showed that the right kidney was enlarged and severely damaged (Fig. 2). Histopathology results for the right kidney were consistent with renal cell carcinoma. The patient was advised to undergo radical nephrectomy. However, she refused surgery and was discharged from hospital. On October 10, 2017, the patient expelled two living worms, and presented gross hematuria and loin pain. The worms were blood red in color, approximately 25 cm in length and 5–7 mm in width and tapered at both the anterior and posterior ends. On the following day, one worm was sent to our department for confirmation. Based on the morphological characteristics of the worm, it was identified as *D. renale* (Fig. 3). The patient was advised to take albendazole as treatment. During the treatment, she expelled another 13 worms and some fragments. After that, her symptoms improved over the following days. However, recently, cancer cells spread to the lungs and bones and the patient could not walk.

## Materials and methods

**Figure 4:**
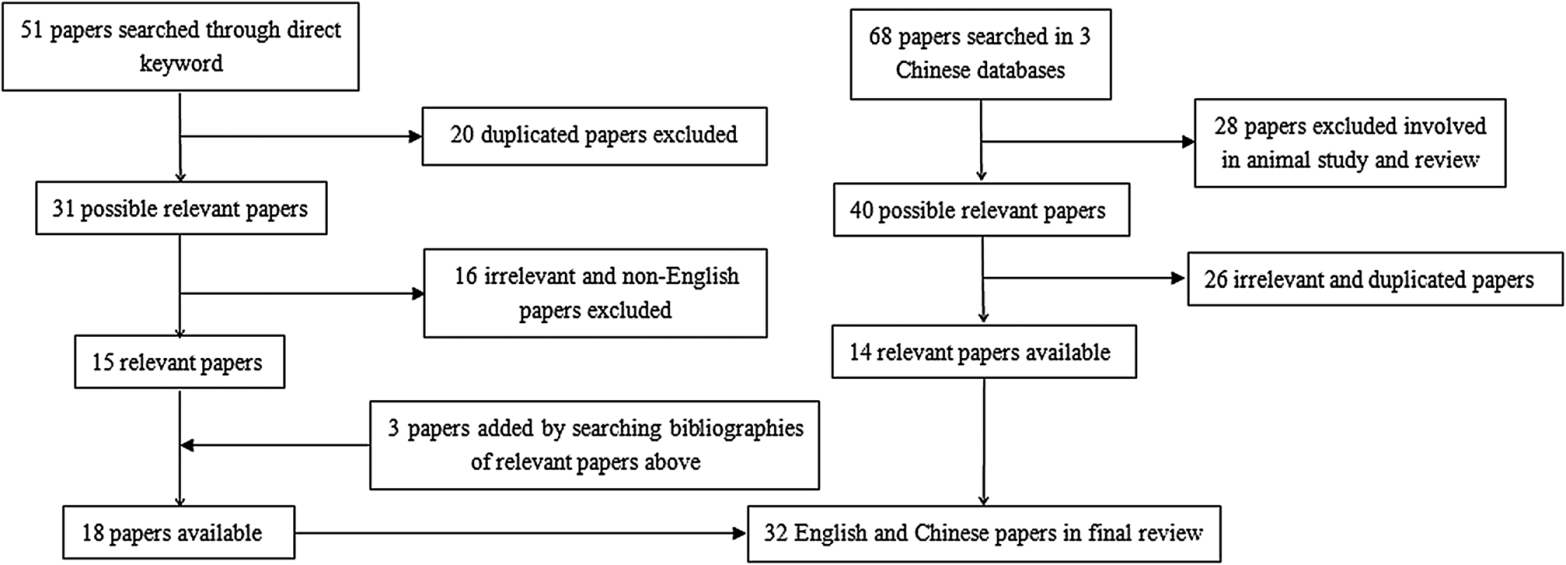
Study selection information regarding human dioctophymiasis.

We present a human case of *D. renale* infection accompanied by renal cancer. Meanwhile, a systematic search was conducted using the following keywords (*Dioctophyma renale* or giant kidney worm and humans) to screen relevant papers published before October 2018 from PubMed (https://www.ncbi.nlm.nih.gov), and the papers were restricted to those published in English (from all years). Likewise, screening Chinese papers (from all years) was also carried out in three Chinese databases (http://www.cnki.net/, http://www.wanfangdata.com.cn/index.html and http://qikan.cqvip.com/). All the titles, abstracts and full texts were examined and reviewed to determine whether the studies described human dioctophymiasis. We excluded duplicate papers and those not for human cases of dioctophymiasis. Additional papers were obtained by searching the reference lists in the papers identified. Our search strategy is illustrated in Figure 4.

## Results and discussion

Based on the search strategy, in the end, 32 papers met our inclusion criteria and were eligible for this review, including 18 and 14 papers searched in PubMed and Chinese databases, respectively. To understand the epidemiological and clinical characteristics of human dioctophymiasis, key information was extracted describing 37 human cases of dioctophymiasis including the present case.

### Epidemiology

#### Geographical distribution

These cases distributed in Asia – China (*n* = 22), Indonesia (*n* = 1), Iran (*n* = 2), India (*n* = 2), Thailand (*n* = 1) and Japan (*n* = 2), in Oceania – Australia (*n* = 1), in Europe – Yugoslavia (*n* = 1), Greece (*n* = 1), and in North America – the United States (*n* = 4) (Table 1). The highest number of human dioctophymiasis cases occurred in China, distributing in 14 provinces (Fig. 1). This might be related to the fact that local inhabitants have poor habits of eating raw or uncooked fish or frogs and drinking unboiled water. In addition, living environments contaminated by *D. renale* eggs in urine from animals can also increase the chances of humans contracting dioctophymiasis. In fact, high infection rates of *D. renale* have been reported in some animals, such as 8.45% in dogs and 25.64% in yellow weasels [32, 36].

#### Age and gender distribution

Of 37 patients infected with *D. renale*, ages ranged from 6–92 years with the mean age of 41.95 years. The youngest and the oldest cases occurred in China. The vast majority of patients (91.9%, 34/37) were over 18 years of age. The reason for this age distribution pattern is not clear but may be related to the predominant modes of exposure. Meanwhile, males (59.5%, 22/37) were observed to have a higher infection rate than females (40.5%, 15/37) worldwide. However, in China, male cases (*n* = 10) were approximately equal to female cases (*n* = 12).

#### Source of infection

Freshwater fish and frogs are considered the major sources for *D. renale* infections in humans and mammals [21]. In our analysis, 17 individuals (45.9%) were suspected to have acquired infection with *D. renale* by eating raw or undercooked fish or frogs. Six cases were considered to be related to ingestion of unboiled water contaminated by oligochaetes harboring third-stage larvae, with three of them having a history of eating fish or frogs (Table 1). Adult worms of *D. renale* are often found in a variety of mammals as definitive hosts, including canines, minks, wolves, foxes, jackals, coyotes, skunks, ferrets, weasels, rats, raccoons, wolverines, pumas, cats, seals, pigs, horses, and humans worldwide [21]. In China, to date, at least ten mammal species have been reported to be infected with this worm, including dogs, minks, yellow weasels, brown rats, seals, cattle, pigs, horses, cats and cheetahs [36]. The patient reported here was born in Heihe city in Heilongjiang province and had been living there for over 40 years. She denied eating raw fish and frogs as well as traveling to other regions. However, she had a habit of drinking unboiled water, which might be the etiology of this case.

### Clinical characteristics

Thirty-one (83.8%) out of 37 cases involved the kidneys, with three of them showing bilateral kidney invasion [4, 14, 19]. The right kidney was invaded more frequently than the left one, which was considered to be the result of a close association with the stomach [19]. The patients suffering from dioctophymiasis usually presented with loin pain (59.5%, 22/37) and hematuria (59.5%, 22/37), which may result from migration of worms [10]. Occasionally some patients may present with fever, anemia, abdominal pain, and weight loss as well as frequent and urgent urine and retention of urine. Urinalysis demonstrated pyuria and proteinuria (Table 1). In extreme circumstances, death could occur due to dioctophymiasis. In Indonesia, one patient with dioctophymiasis finally died of deterioration in his general condition and sepsis after nephrectomy. The patient had expelled in total 25 worms with some of them being fragmented and the histopathology result of the infected kidney was epidermoid cancer [24]. The authors speculated that renal cancer might have been caused by metaplasia of the renal parenchymal cells from parenchymal mass destroyed by the worms. In fact, there is increasing evidence that chronic inflammation of parasitism predisposes to malignancy of the urinary tract [3], such as schistosomiasis, visceral leishmaniasis and malaria caused by *Schistosoma haematobium*, *Leishmania donovani* and *Plasmodium falciparum*, respectively [29]. Kuehn et al. reported another fatal case of a 71-year-old American male with dioctophymiasis, who simultaneously suffered from renal cell carcinoma; however, the authors thought there was no association between them [17]. The third fatal case of human dioctophymiasis occurred in China [19]. A 51-year-old woman was confirmed to have bilateral kidney invasion of *D. renale* based on the results of a CT scan and a history of 39 fragments of red worms expelled. She finally died of bilateral renal function failure. This might be associated with the progressive inflammatory reaction and fibrosis caused by *D. renale.* The patient described here was initially diagnosed with renal cell carcinoma. Currently, it is unclear whether there is a relationship between *D. renale* infection and occurrence of renal cell carcinoma.

Ectopic parasitism of *D. renale* in humans often occurred in subcutaneous tissue in the form of larvae: two cases in the abdominal wall from Japan to the USA [11, 27], two cases in the chest wall from the United States to Thailand [1, 2] and one case in the thigh from Japan [28]. Inflammatory nodules or papules could be observed in the affected region, with the papules being associated with itching [27, 28]. Besides that, there was one case in the retroperitoneal cavity in the form of adult worms in the United States, and the patient had low fever and leukocytosis [25].

### Diagnosis and treatment

Current clinical diagnosis of dioctophymiasis is based on the morphological characteristics of *D. renale* eggs or worms in a patient’s urine or histopathological sections after exploratory surgery [14]. The present data demonstrate that the majority (25/37, 67.57%) of cases were diagnosed through analysis of urine from adult patients. Of course, obtaining patient history (i.e., if the patient has consumed raw or undercooked freshwater fish or frogs) is an important first step, coupled with radiological examinations to search for enlarged or calcified kidneys. Five cases of subcutaneous nodules were all confirmed by identification of *D. renale* larvae based on histopathological observation [1, 2, 11, 27, 28], with one of them being further identified by PCR amplification and sequencing of the small subunit (SSU) rRNA gene and the mitochondrial cytochrome c oxidase subunit 1 (*cox I*) gene [27].

Currently, there is no strictly defined therapeutic approach. The only known means is surgical removal of worms and the damaged portion of the kidneys [21, 24]. Nephrectomy is generally considered extreme for human cases [14]. The use of anti-helminth drugs has not yet been evaluated as the proper course of action to treat *D. renale* infection. However, a patient was cured effectively with ivermectin after two regimens of this medicine [14]. In China, three cases received treatment with albendazole, and no adult worms and eggs were observed later [20, 33, 34].

## Conclusions

This is the first retrospective study of human dioctophymiasis worldwide. Comprehensive epidemiological and clinical characteristics of this parasitic disease are presented here, enhancing the understanding of this parasitic disease. The clinical signs of human dioctophymiasis are not suggestive of dioctophymiasis especially in the early stage. Thus, in clinical practice, when patients present with unexplained loin pain, hematuria and kidney damage, dioctophymiasis should be considered. Although there is no clear relationship between *D. renale* infection and occurrence of renal cell carcinoma, in patients with renal cell carcinoma, it might be useful to also rule out *D. renale* infection. In prevention of human dioctophymiasis, the simplest and most effective way is to avoid eating raw or undercooked fish or frogs as well as drinking unboiled water. Therefore, it is necessary to strengthen public awareness and education to make people aware of the severity of this parasitic disease and of the importance of healthy eating and drinking habits.
